# Knockout of a gene encoding a Gγ protein boosts alkaline tolerance in cereal crops

**DOI:** 10.1007/s42994-023-00106-8

**Published:** 2023-07-02

**Authors:** Peitong Wang, Jian Feng Ma

**Affiliations:** Institute of Plant Science and Resources, Okayama University, Chuo 2-20-1, Kurashiki, 710-0046 Japan

**Keywords:** Alkaline stress, Sorghum, H_2_O_2_, Aquaporin, G protein

## Abstract

Sorghum is highly tolerant to alkaline stress, but the underlying mechanisms are not well understood. Here, based on genotypic difference in alkaline tolerance of sorghum, it was found that AT1 (Alkaline tolerance 1) encoding a G protein is involved in alkaline tolerance through negatively modulating the phosphorylation level of PIP2, an aquaporin with transport activity for H_2_O_2_. Knockout of AT1 releases its inhibition of PIP2, thereby resulting in an increased transport of H_2_O_2_ from the cytosol into the apoplast, subsequently boosting alkaline tolerance.

It is predicated that the global population will reach 9 billion by 2050 (Godfray et al. 2010). Therefore, there is an urgent demand for increasing crop production to feed the increasing population. Given the fact that expansion of lands suitable for crop production is limited, increasing crop productivity will be a key issue, especially in regions with marginal soils (problem soils). Approximately 70% of arable soils are considered to be problem soils, where crop productivity is very low due to the presence of various constraints for crop growth. These typical problem soils include acidic, saline, and sodic soils; therefore, a major contribution to solving food problem would be to boost crop productivity on these potentially arable soils.

It is estimated that sodic soils cover ~ 600 million hectares of land, worldwide, and this area is increasing due to climate change (https://www.fao.org/3/i5199e/i5199e.pdf). Such sodic soils are characterized by poor soil structure, high pH, elevated salt content, and low nutrient concentrations (Li and Li 2022). The main salts in sodic soil are NaHCO_3_ and Na_2_CO_3_, and the pH value is generally higher than 8.5, and even as high as 10–11. Therefore, plants have to cope with both salinity and alkaline (high pH) stress when grown on sodic soils, which constitute serious constraints for most crops.

However, there is a great natural variation in the tolerance to alkaline stress between species and varieties within a species. Sorghum, originating from Africa, has higher alkaline tolerance, compared with other cereal crops. Some sorghum varieties can survive in a soil pH value as high as 10 (Griebel et al. 2021). In a recent Science paper, Zhang et al. (2023), reported their findings on a sorghum gene, *SbAT1* (for *Alkaline Tolerance 1*), involved in alkaline tolerance. Furthermore, they established that this *AT1* gene is also conserved in other cereal crops, including rice, maize, millet, and wheat (Sun et al. 2023; Zhang et al. 2023).

The authors first established a screening system to mimic alkaline stress, in which they incorporated 75 mM mixed alkali salts (62.5 mM NaHCO_3_ and 12.5 mM Na_2_CO_3_) at pH 9.2 to 9.4. Based on genotypic difference in sorghum alkaline tolerance (relative survival rate), the authors performed a genome-wide association study (GWAS), using a sorghum association panel. As a result, a major locus, *SbAT1* on chromosome 1, was detected and the underlying gene encoded an atypical G protein γ (Gγ) subunit. *SbAT1* is a homolog of a previously reported rice *GS3* gene (*Grain Size* 3) (Mao et al. 2010), which has also been reported as *SbGC1* (*Glume Coverage 1*) (Xie et al. 2022).

Membrane-bound G proteins are well studied and play diverse roles in development and environmental interactions. They are heterotrimeric proteins bound to guanine nucleotides and consist of three subunits: G protein alpha (Gα), beta (G_β_), and gamma (Gγ). In plants, the Gγ subunit has been reported to be involved in signal perception, transduction, and regulation of downstream effectors (Pandey 2019). However, this is the first time that this subunit has been shown to be involved in alkaline tolerance.

Based on sequence variation of *SbAT1*, it was found that the variation associates with differences in alkaline tolerance. Two typical haplotypes were identified: Hap1 and Hap2, in different accessions. In contrast to Hap1, carrying intact *SbAT1* (wild-type), Hap2 had a 5-base pair frame shift (GTGGC) insertion in the fifth exon. This insertion results in a premature stop codon, forming a C-terminal truncated protein. The Hap1 accessions exhibited much higher alkaline tolerance compared with those with the Hap2 haplotype. Based on these results, normally one could speculate that *SbAT1* is a gene involved in alkaline tolerance in sorghum.

However, the story is different! When *SbAT1* was knocked out, the alkaline tolerance was increased, rather than undergoing the anticipated decrease, whereas over-expression of *SbAT1* actually increased alkaline sensitivity (Zhang et al. 2023). Similar results were also obtained in rice, maize, millet, and wheat (Sun et al. 2023; Zhang et al. 2023). These findings indicate that SbAT1 acts to negatively regulate alkaline tolerance in cereal crops with small grains.

To resolve this puzzle, Zhang et al. identified the proteins interacting with SbAT1, with the help of IP-MS. Among the proteins detected, they identified a number of aquaporins, especially PIP2;1/PIP2;2. Aquaporins are well known to transport water, but some members are also able to transport small neutral molecules, such as glycerol and silicic acid (Ma et al. 2006; Maurel et al. 2008). Furthermore, some aquaporin members have been reported to be involved in cellular ROS homeostasis (Bienert and Chaumont 2014; Tong et al. 2019; Varadaraj and Kumari 2020). To test whether PIP2 is involved in this alkaline tolerance trait, a rice *PIP2* knockout line was generated, which resulted in decreased alkaline tolerance. Further analysis revealed that H_2_O_2_ accumulated to higher levels in the *PIP2* mutants, over-expression lines of *AT1*, and wild-type plants, compared with various *AT1* knockout lines (Zhang et al. 2023). These results indicate that AT1 negatively regulates alkaline tolerance, via PIP2.

Phosphorylation of PIP2 is required for its activity. Comparison of PIP2 phosphorylation levels showed that they were higher in the *AT1* knockout lines in rice and sorghum compared with the wild-type, over-expression lines, and truncated mutants. Furthermore, in rice roots, more ROS was detected in the cytosol, but less in the apoplast in *AT1* over-expression lines, compared with the wild type, but knockout of *AT1* resulted in a reduced ROS accumulation under alkaline stress conditions.

Based on their findings, the authors proposed a model for AT1-regulated alkaline tolerance. Alkaline stress induces an accumulation of ROS (H_2_O_2_), in the cytosol, which then causes cellular oxidative stress, leading to cell death. Phosphorylated PIP2, localized at the plasma membrane, functions in exporting H_2_O_2_ from the cytosol into the apoplast to reduce oxidative stress (Fig. 1). AT1 bound with G_β_ negatively modulates the phosphorylation of PIP2, although the exact mechanism is unclear. Knockout of *AT1* releases the inhibition of PIP2 phosphorylation, resulting in an increased export of H_2_O_2_ from the cytosol, which is associated with increased alkaline tolerance. By contrast, over-expression of *AT1* decreases PIP2 phosphorylation, resulting in over-accumulation of H_2_O_2_. The observed increased sensitivity of truncated AT1 is probably attributed to the inhibitory role of its C-terminal domain, which is necessary for protein degradation (Zhang et al. 2023).

**Fig. 1:**
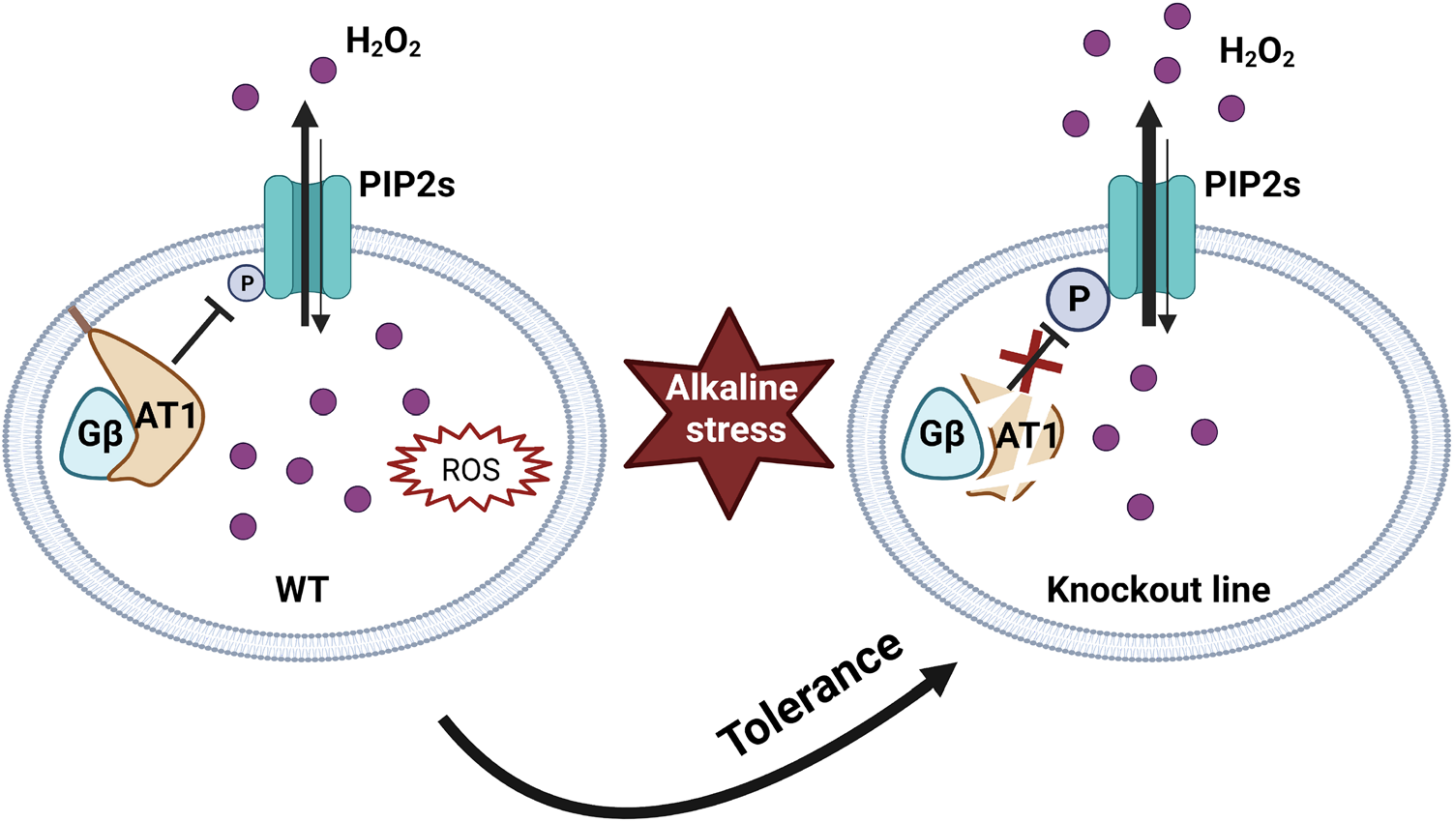
Scheme for AT1-mediated alkaline tolerance in cereal crops. Alkaline stress causes accumulation of H_2_O_2_ in cells, which is then transported, by the phosphorylated form of PIP2, from the cytosol into the apoplast. The G_γ_ subunit, AT1 with G_β_, negatively modulates the phosphorylation level of PIP2, leading to alkaline sensitivity. Knockout of AT1 releases its inhibition of PIP2, thereby resulting in an increased transport of H_2_O_2_ from the cytosol into the apoplast, subsequently boosting alkaline tolerance

A field test was performed in sodic soil and, here, knockout lines of *AT1* showed better growth performance than either the wild-type or over-expression lines in rice, sorghum, maize and millet (Zhang et al. 2023). Importantly, grain yield was increased by more than 20%–28% in the knockout lines, depending on species.

Currently, a number of salt tolerance genes have been identified and applied for breeding (Munns et al. 2012), but less is known on alkaline tolerance in plants. Interestingly, AT1 is not highly involved in salt tolerance, as only minor differences in growth were observed between wild-type and knockout lines exposed to salt stress, at neutral pH (Zhang et al. 2023). Therefore, identification of AT1 by Zhang et al. (2023) sheds light on how plants can overcome alkaline stress, and this important discovery offers promising strategies for breeding crops with alkaline tolerance.

Sorghum is tolerant to alkaline stress, but it is a puzzle as to why, during evolution, the function of AT1 has not been lost to increase its ability to grow under alkaline stress conditions. One possibility is that AT1, as a subunit of the G protein complex, plays multiple roles in growth and development.

In sodic soils, low nutrient availability, due to high pH, is also a limiting factor for crop production. It will also be important for future studies to identify genes involved in nutrient acquisition and utilization, under alkaline stress conditions. Pyramiding these genes with AT1 could offer a strategy for improving crop productivity on sodic soils.

## Data Availability

Data sharing not applicable to this article as no datasets were generated or analysed during the current study.
